# Identification of an antibiotic from an HTS targeting EF-Tu:tRNA interaction: a prospective topical treatment for MRSA skin infections

**DOI:** 10.1128/aem.02046-24

**Published:** 2024-12-23

**Authors:** Wlodek Mandecki, Maxim Chudaev, Wenjuan Ye, Amy Q. Wang, Kenneth J. Wilson, Xin Xu, Jisun Kim, Dane Parker, David Alland, Pradeep Kumar, Barry Li, Jason H. Yang, Barry Kreiswirth, Jose R. Mediavilla, Juan J. Marugan, Mark J. Henderson, Emanuel Goldman

**Affiliations:** 1 Department of Microbiology, Biochemistry, & Molecular Genetics, Rutgers New Jersey Medical School 5970 , Newark, New Jersey, USA; 2 National Center for Advancing Translational Sciences (NCATS), National Institutes of Health (NIH) 390834 , Bethesda, Maryland, USA; 3 Department of Pathology, Immunology and Laboratory Medicine, Center for Immunity and Inflammation, Rutgers New Jersey Medical School 5970 , Newark, New Jersey, USA; 4 Public Health Research Institute, Rutgers New Jersey Medical School 12286 , Newark, New Jersey, USA; 5 Department of Microbiology, Biochemistry & Molecular Genetics, Ruy V. Lourenço Center for Emerging and Re-Emerging Pathogens, Rutgers New Jersey Medical School 12286 , Newark, New Jersey, USA; 6 Center for Discovery & Innovation, Hackensack Meridian Health, Nutley, New Jersey, USA; Michigan State University, East Lansing, Michigan, USA

**Keywords:** *Staphylococcus aureus*, ternary complex in protein synthesis, FRET, topical antibiotic for Gram-positive pathogens, (R,R)-tetrahydrochrysene

## Abstract

Because of the urgent need for new antibiotics to treat drug-resistant bacterial pathogens, we employed an assay that rapidly screens large quantities of compounds for their ability to interfere with bacterial protein synthesis, in particular, the delivery of amino acids to the ribosome via tRNA and elongation factor Tu (EF-Tu). We have identified a drug lead, named MGC-10, which kills Gram-positive bacteria, including methicillin-resistant *Staphylococcus aureus* (MRSA), with a MIC of 6 µM, while being harmless to mammalian cells *in vitro* in that concentration range. The antibacterial activity of MGC-10 was broad against over 50 strains of antibiotic-resistant samples obtained from hospital infections, where MGC-10 inhibited all tested strains of MRSA. Extensive selection and screening with MGC-10 did not yield any resistant strains, indicating it may have universal antibacterial activity against *S. aureus*. Pharmacokinetics performed in mice suggested that MGC-10 was too toxic for systemic use; however, it appears to have potential as a topical treatment for difficult-to-treat wounds or skin infections by Gram-positive pathogens such as MRSA. In a mouse skin-infection model with MRSA, MGC-10 performed as well or better than the present topical drug of choice, mupirocin. MGC-10 showed little, if any, accumulation in the livers of topically treated mice. These results bode well for the future use of MGC-10 in clinical application as it could be used to treat a broad range of *S. aureus* skin infections that are resistant to known antibiotics.

**IMPORTANCE:**

There is a critical need for new antibiotics to treat bacterial infections caused by pathogens resistant to many if not all currently available antibiotics. We describe here the identification of a prospective new antibiotic from high-throughput screening of a chemical library. The screening was designed to detect the inhibition of formation of a complex required for bacterial protein synthesis in all bacteria, the “ternary complex,” comprised of elongation factor Tu (EF-Tu), aminoacyl-tRNA, and GTP. The inhibitory compound, renamed MGC-10, was effective against all Gram-positive bacteria, including a wide variety of methicillin-resistant *Staphylococcus aureus* (MRSA) strains. Although apparently too toxic for systemic use, the compound was safe and effective for topical use for treating skin infections in a mouse model. No resistance to the compound has been detected thus far, suggesting the potential to develop this compound for topical use to treat infections, especially those caused by pathogens resistant to existing antibiotics.

## INTRODUCTION

Ternary complex formation between elongation factor Tu (EF-Tu), aminoacylated tRNA (aa-tRNA), and GTP is a crucial step of protein biosynthesis for delivery of amino acids to the growing protein chain by bringing aa-tRNA into the codon-programmed A-site of the ribosome. Although more than half of all antibiotics target bacterial protein synthesis, there are no antibiotics in clinical use that target this step. A few known inhibitors of this step work *in vitro* (kirromycin, factumycin, and others) but are not clinically effective against Gram-negative bacteria because they do not enter bacterial cells or are rapidly excreted (1). They are also not in use for Gram-positive pathogens, possibly because of difficulty in achieving therapeutic concentrations *in vivo*. LFF571, a derivative of GE2270A, showed antimicrobial activity against Gram-positive pathogens but was not pursued for clinical use (2). Hence, ternary complex formation remains a relatively unexplored molecular target with therapeutic potential for treating bacterial infections.

**Fig 1:**
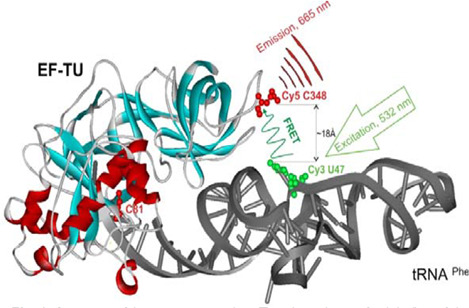
Ternary complex involving EF-Tu and tRNA with two engineered fluorescent labels, Cy3 and Cy5 for the FRET assay (3).

There is ample structural information on the EF-Tu:tRNA ternary complex. A simple search “EF-Tu” in the Protein Data Bank yields over 1,000 structures, many of which include tRNA. Data originate from X-ray crystallography studies as well as cryo-electron microscopy and include high-resolution structures of the EF-Tu:tRNA complex by itself or presented in the context of the ribosome structure. These structures offer detailed views of the EF-Tu:tRNA complex, illustrating how EF-Tu interacts with tRNA and GTP/GDP and providing a framework for understanding the mechanism of tRNA delivery to the ribosome during protein synthesis. Some of the structures include small molecules bound to EF-Tu, and a subset of them include inhibitors of the EF-Tu interaction with tRNA, for example, GE2270A or kirromycin and also GTP and GDP analogs. Much of our work was based on Protein Data Bank entry 1OB2, depicting a complex of the *Escherichia coli* EF-Tu and Phe-tRNA^Phe^, also from *E. coli* (Fig. 1).

As reported in published papers from our group (3–5), we developed a high-throughput screening (HTS) assay to identify inhibitors of ternary complex formation. In this assay, ternary complex formation results in fluorescence resonance energy transfer (FRET) (Fig. 1); inhibitors of ternary complex formation, identified by their ability to inhibit FRET, are therefore potential antibiotics and subject to further testing.

The form of *E. coli* EF-Tu that was used in the assay was designed specifically through advanced protein engineering and carries three mutations that allow for site-directed fluorescence labeling of the mutant protein without limiting the biological activity of the protein *in vitro* (3, 5). The modified EF-Tu and an *E. coli* tRNA molecule specific for the amino acid phenylalanine (tRNA^Phe^) are both fluorescently labeled, with Cy5 and Cy3, respectively. A naturally occurring modified nucleotide at position 47 of tRNA^Phe^ from *E. coli* was chosen for labeling the tRNA. The labeling does not change biological properties of this tRNA *in vitro* (6), and labeled tRNAs have been used in many successful translation experiments. The tRNA^Phe^ we are using is also aminoacylated.

Upon ternary complex formation, FRET occurs when Cy3 is excited at 532 nm; an increased Cy5 fluorescence intensity is then observed at 665 nm. The HTS assay screens for inhibitors of FRET, presumably due to the prevention of ternary complex formation. This type of bioassay is considered a homogeneous assay and is favored in HTS because it is an addition-only, mix-and-read assay, only requiring mixing of the reagents (aa-tRNA:EF-Tu complex, GTP) and the compound being tested, followed by measuring fluorescence intensity at 665 nm.

A screen of the LOPAC-1280 (Library of Pharmacologically Active Compounds) chemical library yielded a number of hits, one of which seemed promising and was subjected to further testing. This compound, (R,R)-tetrahydrochrysene, is a known estrogen receptor ligand (7), with no prior reports of antimicrobial activity. After identifying (R,R)-tetrahydrochrysene in the screen, further testing showed the compound could inhibit Gram-positive bacteria including numerous naturally occurring isolates of MRSA. Because EF-Tu has a very large number of molecular partners in its normal function, we hypothesized that antibiotic resistance to this compound would be difficult and rare; thus far, this appears to be confirmed, as we were not able to find any resistant bacteria after an extensive effort to select for resistance.

Renamed MGC-10, the compound applied topically was at least as effective as the current drug of choice, mupirocin, in a mouse skin infection model. Resistance to mupirocin is becoming an increasing concern (8). Several naturally occurring isolates of mupirocin-resistant *Staphylococcus aureus* were all sensitive to MGC-10. Pharmacokinetic studies in mice showed some toxicity of MGC-10 when administered systemically; it is unusually metabolically stable, accumulating in the liver, and therefore does not appear to be suitable for systemic use. However, very little compound was found in the liver subsequent to topical treatment. Therefore, MGC-10 is a prospective new topical treatment for MRSA skin infections, especially those resistant to antibiotics currently in use.

## RESULTS

### EF-Tu high-throughput assay development and LOPAC1280 library pilot screening

**Fig 2:**
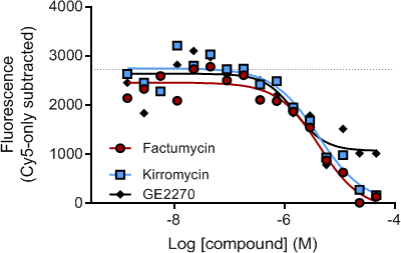
Inhibition of FRET by known inhibitors of EF-Tu; M, molarity. Shown is a representative experiment, which was repeated.

The FRET-based assay to measure EF-Tu:tRNA^Phe^:GTP ternary complex formation was miniaturized to a 1,536-well format. Assay performance, as calculated by Z’ factor (9), was favorable for the Envision plate reader, with a value of Z = 0.4 (Fig. S1). Factumycin, kirromycin, and GE2270, known inhibitors of the ternary complex, were tested as control compounds for the assay. Each showed inhibition of FRET signal with an EC_50_ between 1 and 5 µM (Fig. 2).

Prior to screening, the stability of the reagents and compatibility with HTS were examined by preparing Cy5-EF-Tu and Cy3-tRNA working solutions and incubating them at room temperature for 24 h. FRET and inhibition by factumycin and kirromycin were detectable at each time point, supporting stability of the assay, particularly up to 4 h (Fig. S2). The assay performance with varying incubation time after addition of the Cy3-tRNA component was also assessed, using factumycin, kirromycin, and GE2270 as controls. Compound inhibition profiles were similar from 4 to 12 min but diminished with a longer incubation of 45 min (Fig. S3).

**Fig 3:**
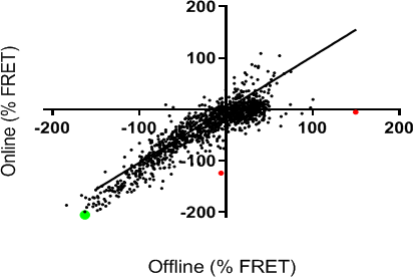
LOPAC1280 results for the offline and online assays. FRET values were normalized to DMSO controls (0%) and 40 µM kirromycin (−100%). Red dots represent compounds that showed large discrepancies between the two methods. The green dot represents MGC-10.

Next, an experiment was designed to assess assay performance using a fully automated procedure (NCATS robotic HTS platform). The LOPAC1280 library was selected for testing using the manual assay (“offline,” described above) versus a fully automated procedure (“online”). The control compound kirromycin performed similarly using both procedures (Fig. S4). The results demonstrated substantial agreement for LOPAC testing for both methods (using an Envision plate reader to detect FRET in both cases), with a final compound concentration of ~38 µM. Fluorescence values were normalized to dimethyl sulfoxide (DMSO) controls (0%) and 40 µM kirromycin (100%), with percent activity derived using software developed at NCATS (http://tripod.nih.gov/curvefit). Compounds that showed large discrepancies between the two methods are shown in red (Fig. 3).

### Identification of a compound having antibiotic activity

**Fig 4:**
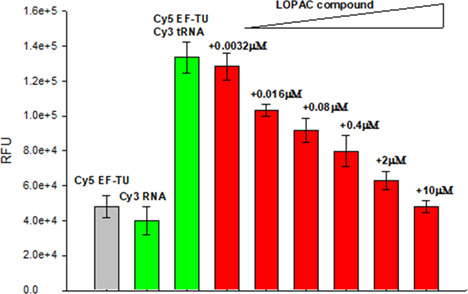
Secondary FRET assay. Controls are represented by two bars on the left side. Cy5 acceptor fluorescence intensity was significantly diminished in the presence of the inhibitor of the reaction, MGC-10. The gray and green bars represent controls of each dye alone attached to EF-Tu or tRNA, respectively (background fluorescence), and the two dyes together without MGC-10 added (FRET). The red bars show the extent of fluorescence in the presence of increasing amounts of MGC-10.

The highest-scoring compounds picked up by the HTS for inhibition of FRET were selected and provided to Rutgers for follow-up studies to determine whether they were *bona fide* EF-Tu:tRNA modulators or fluorescence artifacts. They were tested in zone inhibition assays against *E. coli* and *Bacillus subtilis* (Fig. S2). This led to the identification of (R,R)-tetrahydrochrysene as a top active compound by both methods (Fig. 3, green circle). It has a molar mass of 320.432 g·mol^−1^. Its formula is C22H24O2. Information about (R,R)-tetrahydrochrysene is available from several sources: CAS #: 138090–06-9; PubChem CID: 446849; ChemSpider: 394097; and ChEBI: CHEBI:42371. A dose-dependent activity in the FRET assay was confirmed, with an IC50 (half maximal inhibitory concentration) of 0.17 µM (Fig. 4). The compound did not alter FRET signal in an oligonucleotide-based counterscreen, indicating that it was not a fluorescent artifact (Fig. S6).

**Fig 5:**
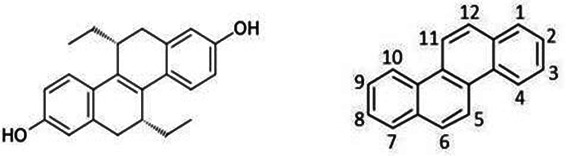
(Left) (R,R)-tetrahydrochrysene chemical structure. (Right) Numbering of carbon atoms in chrysene.

The full chemical name of (R,R)-tetrahydrochrysene is (R,R)−5,6,11,12-tetrahydrochrysene-2,8-diol (Fig. 5). This compound belongs to the class of organic compounds known as tetrahydrochrysenes. They are characterized by a chrysene skeleton that has been hydrogenated in specific positions, and they also feature hydroxyl groups at the 2 and 8 positions.

The compound has a known biological activity: it is a non-steroidal, selective estrogen receptor ligand; agonist at ERα receptor, and antagonist at ERβ receptor (10).

**Fig 6:**
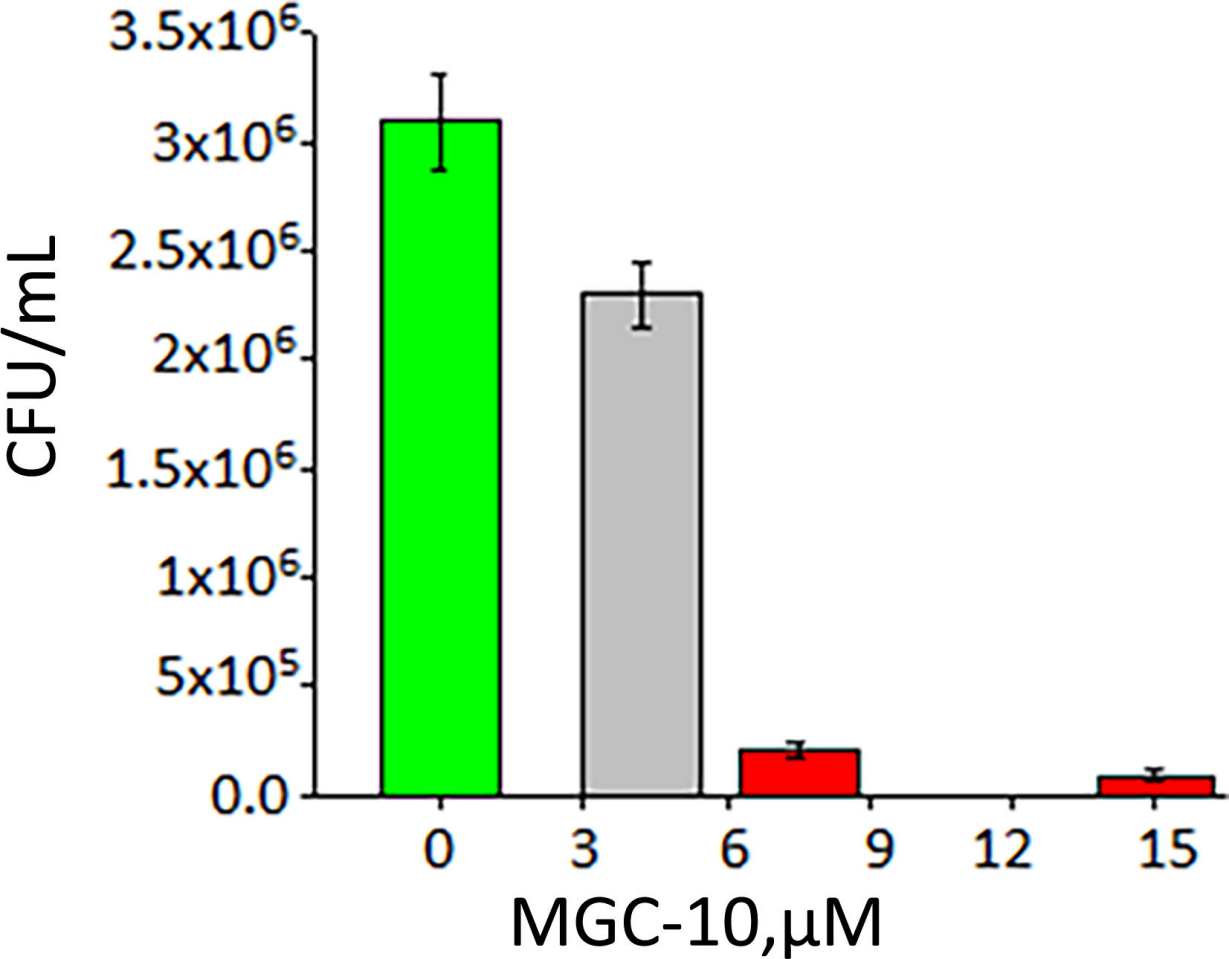
Inhibition of *B. subtilis* growth by MGC-10. The green bar represents growth in the absence of an inhibitor. The red bars represent significant growth inhibition by MGC-10.

In the presence of 6 µM (R,R)-tetrahydrochrysene, referred to as MGC-10 for the remainder of this manuscript, we observed a 97% inhibition of growth of *B. subtilis* (Fig. 6). No inhibition was seen in the case of *Pseudomonas aeruginosa* (Table 1) and *E. coli* (not shown).

**TABLE 1:** MIC values of MGC-10 in several strains

Strain name	MIC (μM)	Cell type
*S. aureus mecA +* (MRSA) (ATCC 43300)	6.25	Gram-positive
*S. aureus* (ATCC 25923)	6.25	Gram-positive
*S. aureus* (biofilm) (ATCC 35556)	6.25	Gram-positive
*P. aeruginosa* (ATCC BAA47)	>100	Gram-negative
*K. pneumoniae* (ATCC 43816)	>100	Gram-negative
*A. baumannii* (ATCC 17988)	>100	Gram-negative
*M. tuberculosis* H37Rv (ATTC 27294)	100	Acid-fast
Vero (ATCC CCL-81)	50	Mammalian

MGC-10 was tested against a few other strains including MRSA and continued to inhibit Gram positives and not inhibit Gram negatives (Table 1). It was also tested for cytotoxicity against Vero mammalian cells, where it showed no cytotoxicity in the concentration range in which it inhibited sensitive bacteria (Table 1).

**Fig 7:**
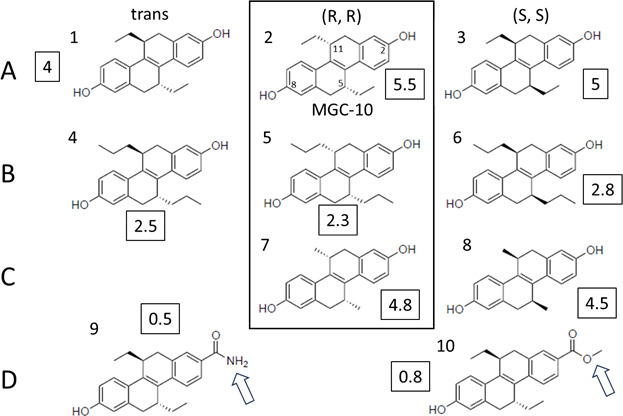
Testing of antibacterial properties of nine derivatives of (R,R)-tetrahydrochrysene (MGC-10). The boxed numbers represent the thickness in mm of zones of inhibition around filter disks containing the respective compounds (see Materials and Methods).

Nine analogs of MGC-10 were kindly provided by Dr. John Katzenellenbogen (University of Illinois Urbana-Champaign). A zone inhibition assay was performed on each of the nine compounds provided. The zone diameter is presented in Fig. 7 as the key biological characteristic of the compounds. The compound with the highest relative antibacterial activity was found to be MGC-10.

### Characterization of MGC-10 *in vitro*

**Fig 8:**
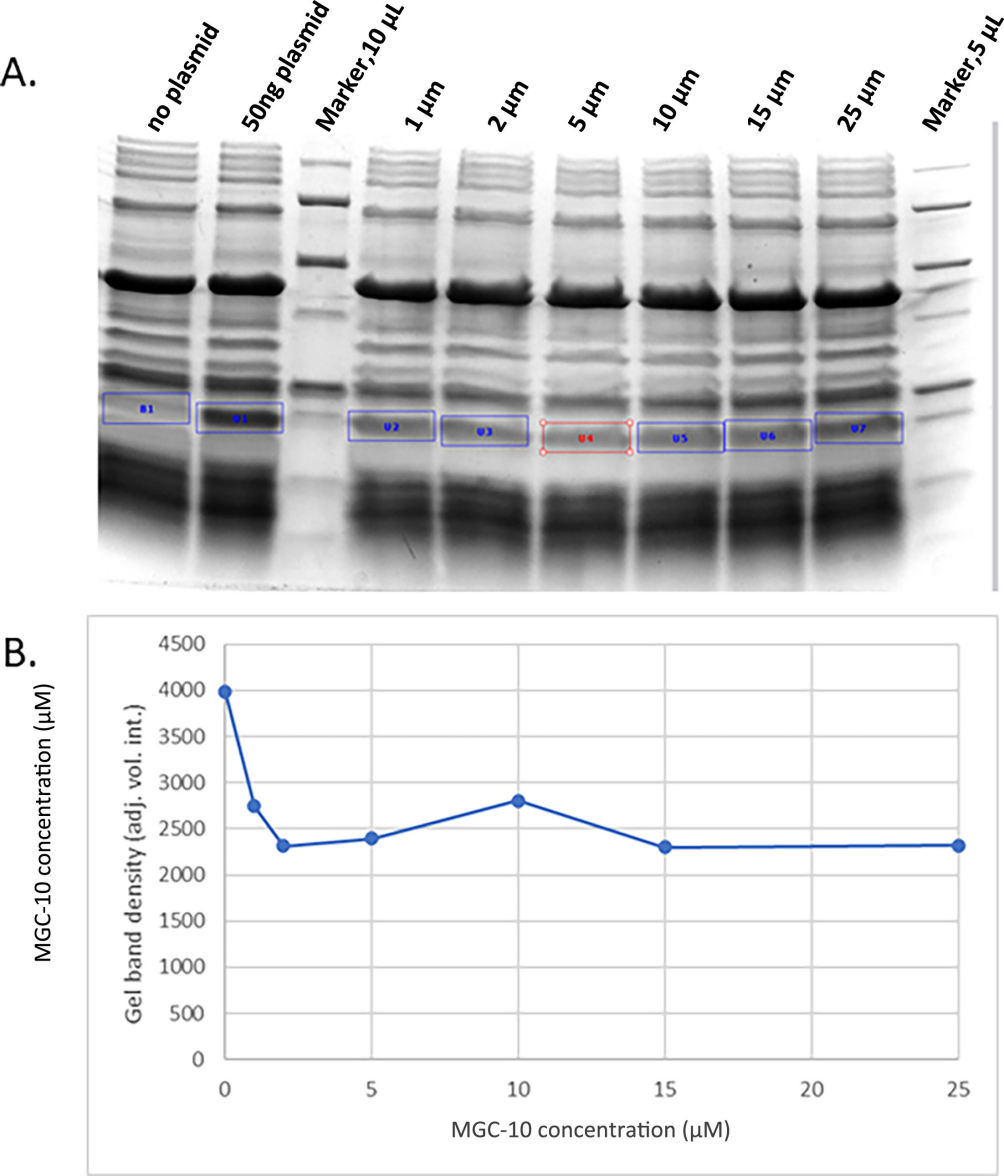
Effects of varying concentrations of MGC-10 on the efficiency of ribosomal translation *in vitro*. (**A**) Coomassie blue staining of sodium dodecyl sulfate polyacrylamide gel electrophoresis (SDS-PAGE) following *in vitro* translation with the PURExpress translation kit (New England Biolabs). The protein synthesized (Dihydrofolate reductase) in the reaction is shown in the small rectangles superimposed on the gel. (**B**) Quantification using a BioRad gel scanner and software provided with this scanner. The density of the image in areas highlighted by the small rectangles on the gel was measured and shown as a function of added MGC-10 concentration.

The effects of MGC-10 on ribosomal translation were evaluated by gel electrophoresis in the purified system PURExpress (New England Biolabs). The results are shown in Fig. 8, panels A and B. Even at low MGC-10 concentrations (1 µM and higher), translation efficiency is significantly reduced; however, complete inhibition was not achieved even at the highest concentration tested.

**Fig 9:**
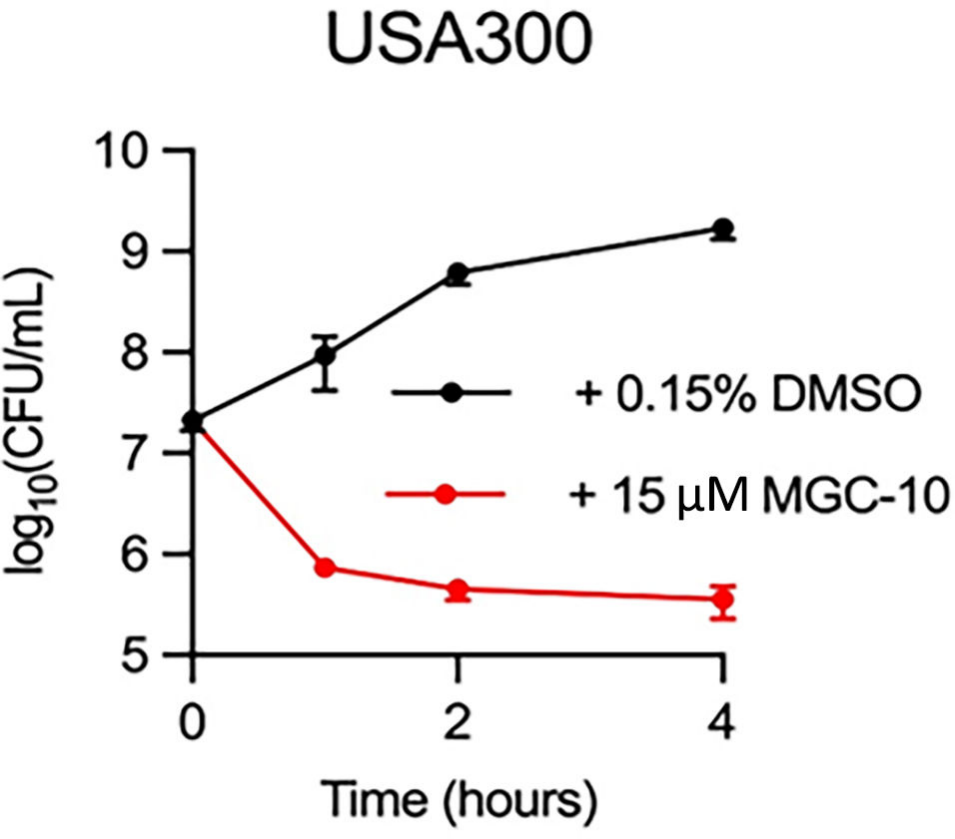
Bactericidal test of MGC-10 on *S. aureus*. Cell survival is shown as a function of time following exposure to MGC-10 or to DMSO (control).

We characterized the effects of MGC-10 in *S. aureus* USA300 and found that MGC-10 exhibits notable bactericidal activity. We know that the effect is likely bactericidal because the density of cells, in CFU/mL, decreased over time as shown in Fig. 9.

A total of 30 methicillin-resistant *S. aureus* (MRSA) strains corresponding to nine different multilocus sequence type (MLST) clonal complexes and having unique resistance phenotypes were selected for testing antibacterial properties of MGC-10, along with a methicillin-susceptible control strain. An additional 23 non-aureus staphylococcal strains corresponding to 14 different species were also tested, along with seven naturally occurring high-level mupirocin-resistant *S. aureus* strains. The results (Table 2) indicate that all strains are inhibited by MGC-10. Both the MIC90 (antibiotic concentration that inhibits 90% of bacterial colonies) and MIC50 (antibiotic concentration that inhibits 50% of bacterial colonies) are 6 µM for 29 of 31 MRSA strains and 18 of 23 other Staphylococcal strains. The remaining seven strains showed an MIC of 12 µM. One of the mupirocin-resistant strains also showed a MIC of 12 µM, but another one showed an MIC of only 1.5, whereas the remaining five strains were either 3 or 6 µM.

**TABLE 2:** Tests of MGC-10 against a panel of *Staphylococcus* strains^*a*^

*Staphylococcus aureus*				Staphylococcal species			
BK strain no.	Clonal complex	MIC	Comments	BK strain no.	Species	MIC	Comments
19484	CC 1	6 µM	USA400	22034	*S. capitis*	12 µM	
23736	CC 1	6 µM	BORSA	24463	*S. capitis*	6 µM	
23735	CC 125	6 µM	BORSA	22945	*S. caprae*	6 µM	
21347	CC 22	6 µM	Linezolid resistant	22939	*S. cohnii*	12 µM	
63193	CC 30	6 µM		21192	*S. epidermidis*	6 µM	Methicillin R
63765	CC 30	12 µM		26974	*S. epidermidis*	6 µM	Methicillin R
26638	CC 398	6 µM	LA-MRSA	26189	*S. gallinarum*	12 µM	
61828	CC 398	6 µM		21148	*S. haemolyticus*	12 µM	
62976	CC 398	6 µM		26191	*S. lentus*	6 µM	
63776	CC 398	6 µM		26639	*S. lentus*	6 µM	Methicillin R
18519	CC 45	6 µM	USA600	26192	*S. lugdunensis*	6 µM	
62861	CC 45	6 µM		28088	*S. lugdunensis*	6 µM	Methicillin R
63777	CC 45	6 µM		24474	*S. pseudintermedius*	6 µM	
18267	CC 5	6 µM	USA800	26190	*S. pseudintermedius*	12 µM	
21346	CC 5	6 µM	Tigecycline resistant	25938	*S. pseudintermedius*	6 µM	
22522	CC 5	6 µM	Vancomycin resistant (*vanA+*)	26194	*S. saprophyticus*	6 µM	
22523	CC 5	6 µM	Vancomycin resistant (*vanA+*)	26195	*S. saprophyticus*	6 µM	
58711	CC 5	6 µM	Rifampin resistant	26199	*S. sciuri*	6 µM	
62961	CC 5	6 µM		26200	*S. sciuri*	6 µM	
62969	CC 5	6 µM		26201	*S. simulans*	6 µM	
62874	CC 59	6 µM		24473	*S. warneri*	6 µM	
63738	CC 59	6 µM		26202	*S. warneri*	6 µM	
13180	CC 8	6 µM	Trimethoprim-sulfamethoxazole resistant	26203	*S. xylosus*	6 µM	
19494	CC 8	6 µM	USA300	73239	*S. aureus*	1.5 µM	Mupirocin R
21189	CC 8	12 μM	MSSA (NCTC 8325)	73242	*S. aureus*	3 µM	Mupirocin R
21209	CC 8	6 µM	Linezolid resistant	73250	*S. aureus*	3 µM	Mupirocin R
46376	CC 8	6 µM	hVISA	73267	*S. aureus*	6 µM	Mupirocin R
46377	CC 8	6 µM	hVISA	73269	*S. aureus*	6 µM	Mupirocin R
59775	CC 8	6 µM	Trimethoprim-sulfamethoxazole resistant	73336	*S. aureus*	12 µM	Mupirocin R
63218	CC 8	6 µM		73337	*S. aureus*	3 µM	Mupirocin R
63341	CC 8	6 µM					

^*a*^ BORSA, borderline oxacillin-resistant *S. aureus*; LA-MRSA, livestock-associated methicillin-resistant *S. aureus*; MSSA, methicillin-sensitive *S. aureus*; hVISA, heterogeneous vancomycin-intermediate resistance *S. aureus*; R, resistant. All strains in the left column were MRSA except for the strain noted as MSSA (21189). The seven mupirocin-resistant *S. aureus* strains in the right column were all MSSA.

We attempted to find spontaneous resistance mutants to MGC-10. Approximately 5 × 10^9^ cells were screened, and no mutants resistant to MGC-10 were found (see Materials and Methods). We also screened an *S. aureus* transposon library for loss-of-function mutations that would lead to resistance but again did not identify a resistant mutant (data not shown).

We tested the stability of MGC-10 *in vitro*. In the presence of both rat and human liver microsomes, no depletion of the compound was observed after 60 min of incubation (data not shown).

### Characterization of MGC-10 *in vivo*

Concentration-time profiles after IP (intraperitoneal injection) administration of 10 mg/kg MGC-10 to mice are shown in Fig. S7. The pharmacokinetic parameters of MGC-10 after 10 mg/kg IP administration are shown in Table 3.

**TABLE 3:** Pharmacokinetic parameters of MGC-10^*a*^

Parameter	MGC-10				
Sample	Units	Plasma	Liver	Lung	Brain
T_last_	h	24	24	7	7
C_last_	ng/mL	28	2,166	733	532
AUC_(0-7h)_	h*ng/mL	150,935	33,693	7,246	4,355
AUC_last_	h*ng/mL	170,508	84,515	7,246	4,355
AUC_inf_	h*ng/mL	170,592	NR	NR	NR
t_1/2_	h	2.1	NR	NR	NR
T_max_	h	0.17	1	0.17	0.17
C_max_	ng/mL	57,050	6,407	2,650	869
AUC_(0-7h)_ ratio (tissue/plasma)			0.22	0.048	0.029

^*a*^ NR, not reportable because AUC (% extrapolated) was >30%.

In-life observations of the mice were as follows: (i) at 48 h post-dose: five mice appeared quiet, alert, and responsive; exhibited hunched postures; and were lethargic. They were provided diet gel for hydration. (ii) At 72 h post-dose: four mice were found dead, and we were unable to collect blood and organs.

We conclude the following: for 10 mg/kg IP administration of MGC-10 in mice, the compound is toxic with four of six animals dead at 72 h post-dose. We observed high MGC-10 concentrations in plasma. Tissue-to-plasma area under the curve (AUC) ratios were 0.22 and 0.048 for liver and lung, respectively. Lower concentrations in liver and lung indicated low Vss (volume of distribution at steady state). MGC-10 did not pass the blood brain barrier.

**Fig 10:**
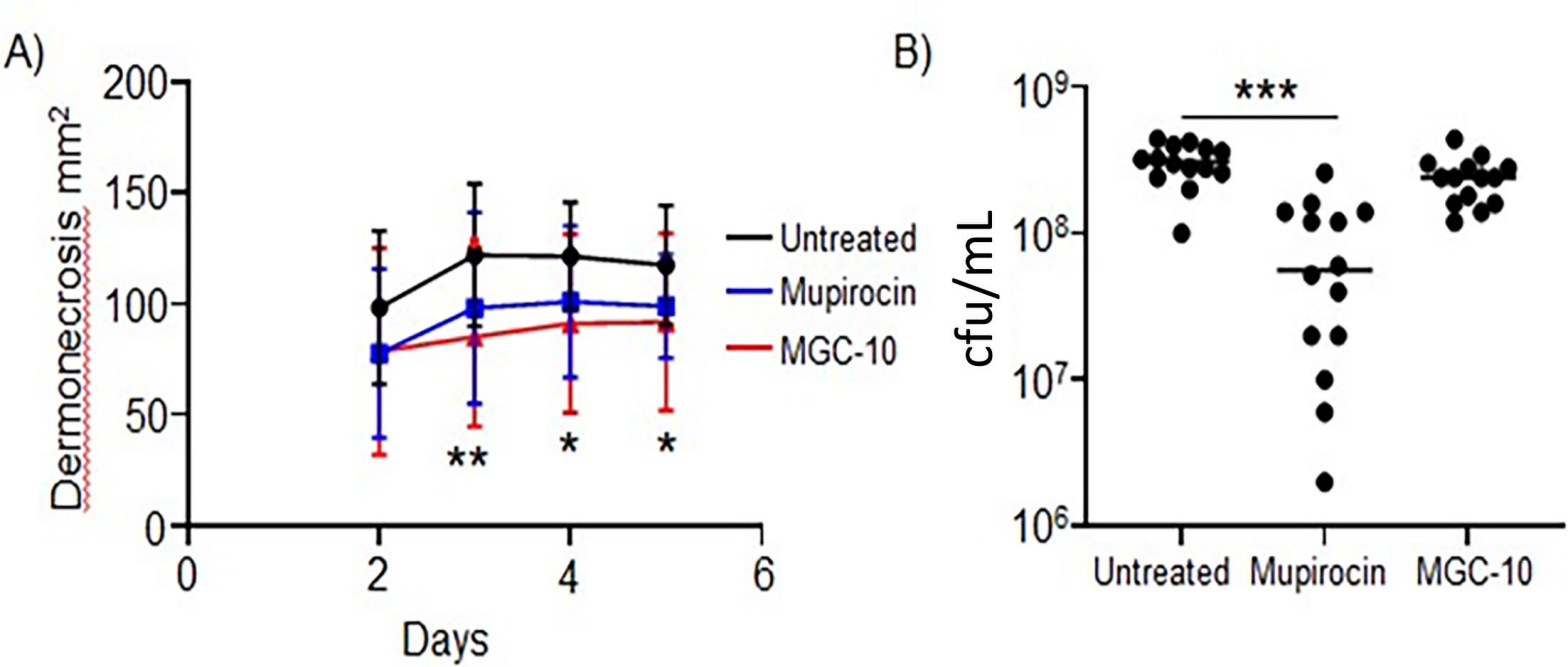
MGC-10 reduces *S. aureus*-induced dermonecrosis. Mice were infected subcutaneously with *S. aureus* USA300 for 5 days. (**A**) Area of dermonecrosis measured over time. Lines display mean and error. Statistical significance between the untreated control (ointment without MGC-10) and MGC-10 treated mice. (**B**) Bacterial concentration in punch biopsies at the conclusion of the experiment. *n* = 18 untreated and *n* = 17 mupirocin and MGC-10. Each point represents a mouse. Lines display median. **P < 0.01 and *P < 0.05.

To determine the efficacy of MGC-10 in an *in vivo* model of infection, we tested its ability to ameliorate a subcutaneous infection of *S. aureus*. We compared the effectiveness of MGC-10 with mupirocin, both as topical preparations. We observed that MGC-10 was able to reduce dermonecrosis as a result of *S. aureus* infection (Fig. 10A). Mupirocin significantly reduced the bacterial burden (Fig. 10B). MGC-10 did show evidence of reduced bacterial burden but this was not statistically significant (*P* = 0.0575). Little if any MGC-10 was found in liver samples of animals treated topically (data not shown).

## DISCUSSION

Because of worldwide frequent and sometimes inappropriate or excessive use of antibiotics, bacterial pathogens resistant to multiple antibiotics have been emerging and have become an increasing threat to human health, as physicians lose their ability to treat these infections. A recent Lancet review estimated nearly 5 million deaths per year are attributable to antibiotic-resistant bacteria, with MRSA as a leading cause (11). The prevalence of MRSA in nasal passages in the US population is estimated at ~2% (12). The annual incidence of MRSA infection in the US is ~70,000 (13). To counter this threat, we are in desperate need of new antibiotics to control resistant pathogens such as MRSA.

In a commentary in the journal *Nature*, an appeal was made to increase emphasis on screening vast libraries of compounds (tens of millions of compounds) to obtain antibiotic drug leads (14). The method described in this study was designed with this type of high-throughput approach in mind and could facilitate the identification of additional drug leads, similar to MGC-10, for the treatment of bacterial infection.

EF-Tu is an ideal target for developing novel antibacterial agents because the protein is essential and highly conserved among bacteria. For example, EF-Tu from *Klebsiella* is almost identical to *E. coli* EF-Tu, showing 97% sequence alignment homology (5). Thus, it is highly likely that results with *E. coli* EF-Tu will be identical for *Klebsiella*. Although *E. coli* is considered a research model organism, it can also be pathogenic. This is especially evident in deadly strains like O157:H7. Urinary tract infections and many diarrheas result from *E. coli* as well. If *E. coli* enters the bloodstream, septic shock and death can ensue. *E. coli*’s close relative, *Klebsiella pneumoniae*, also causes deadly nosocomial infections such as pneumonia. Treating these diseases has become compromised with the recent emergence of carbapenem-resistant “superbugs.”

A homology alignment of protein sequences of EF-Tu from *E. coli* and other bacterial pathogens (including MRSA), eEF-1a (the eukaryotic homolog to EF-Tu), and human mitochondrial EF-Tu demonstrated that there is no identifiable homology to eEF-1a or human mitochondrial EF-Tu (5). Therefore, one can reasonably expect to find inhibitors of bacterial ternary complex formation that do not affect human ternary complex. Indeed, kirromycin and other inhibitors of this step only inhibit bacterial protein synthesis and not eukaryotic protein synthesis *in vitro* (1, 15).

Four families of antibiotics of unrelated structures comprising a total of over 30 members target EF-Tu (16). The prototype chemical compounds for the four families are kirromycin, enacyloxin IIa, pulvomycin, and GE2270A. These are naturally occurring antibiotics derived from *Streptomyces* and related species that block the path leading to presentation of the ternary complex to the ribosome *in vitro*. They are selective against bacterial protein synthesis and do not inhibit eukaryotic protein synthesis. Inhibition of protein biosynthesis by these compounds is based on two different mechanisms: (i) kirromycin and enacyloxin freeze EF-Tu in complex with GDP, such that it sticks to ribosomes and prevents further protein synthesis, and (ii) pulvomycin and GE2270A block formation of ternary complex between EF-Tu, GTP, and tRNA. Although there are different mechanisms of action for the two types of EF-Tu inhibitors, both result in inhibition of FRET in our assay, as shown in Fig. 2. Kirromycin freezes EF-Tu in complex with GDP, and the complex remains bound to the ribosome (16), thereby removing bound EF-Tu from the pool of available molecules since this form of EF-Tu is unable to participate in ternary complex formation. Preventing EF-Tu from being able to bind aa-tRNA will also prevent FRET in our assay.

These antibiotics are not in clinical use for Gram negatives because they do not enter bacterial cells or are rapidly excreted (1). Although permeability appears not to be the problem with Gram positives, many Gram-positive bacteria seem to contain EF-Tu forms that are naturally resistant to levels of kirromycin that could be achieved clinically (17). LFF571, a derivative of GE2270A, showed antimicrobial activity against Gram-positive pathogens but was not developed for clinical use (18).

ET-Tu has to interact with many molecular partners for protein synthesis to be able to function. These include 40 or more different tRNAs and proteins that include EF-Ts and ribosomal proteins. Therefore, developing resistance to EF-Tu inhibitors is expected to be difficult for the pathogen because of the plethora of molecular interactions that could be adversely affected by any mutation. Tests with MGC-10 in *S. aureus* in fact showed resistance is not easily developed; despite intensive efforts, we were unable to find resistant cells.

The HTS of the LOPAC library had a relatively high hit rate, approximately 5%. A hit rate this high requires robust secondary screens to narrow the number of candidate drug leads for further testing. We found that zone inhibition using filter disks on Petri dishes was a relatively quick method to pick up bacterial inhibitors and avoid other compounds that reduced FRET in the HTS but did not inhibit bacterial growth.

The HTS assay is based upon specific interaction between modified EF-Tu and tRNA; however, it is important to note that these FRET partners depend upon interaction with a third component, GTP, to form a ternary complex. This is not a trivial matter, as it is known that the GDP form of EF-Tu does not bind tRNA and needs to be recycled via EF-Ts to the GTP form, which is then functionally active. Therefore, a possible explanation for the high hit rate could be that if any small molecule that binds to EF-Tu (or tRNA for that matter) alters the tertiary conformation of the protein (or tRNA), that alteration might be sufficient to disrupt complex formation, and would score as a hit by preventing FRET, even if the inhibition was not specific to the expected interface between EF-Tu and tRNA. The fact that three components need to dock fairly precisely in ternary complex could make these molecules much more sensitive to allosteric alterations compared with FRET assays involving only binary pairs of partners. There may of course be other reasons for the high hit rate, such as unanticipated quenching.

Regarding structure-activity relationship studies, the small set of analogs evaluated indicated that parts of the MGC-10 molecule responsible for antibacterial activity are the two outside phenyl rings rather than the central part of the molecule, since the *trans*, (R,R) and (S,S) conformations and the two dimethyl derivatives do not vary much in terms of the biological activity in the zone inhibition assay. On the contrary, relatively large changes were seen when the hydroxy groups on the outside phenyl rings were substituted. Further modifications to the MGC-10 molecule could potentially improve potency, efficacy, or pharmacokinetic properties. Furthermore, defining the binding site of MGC-10 within the ternary complex via crystallography could be used to further optimize the inhibitors. We intend to address these questions in the future.

Because the assay was designed to pick up inhibitors of EF-Tu function, it was expected that the mechanism of action of MGC-10 would be to inhibit bacterial translation, and indeed, our results with *in vitro* protein synthesis support this. The inhibition of translation was not complete and was observed at lower concentrations *in vitro* compared with the MIC50 *in vivo*, which is also an order of magnitude greater than the IC50 for the *in vitro* FRET assay. However, these are very different assays. The MIC50 requires the compound to enter cells from outside and then requires the compound to find its target in a very messy and crowded intracellular environment. The IC50 is from a purified *in vitro* system, and not whole living cells. The IC50 is also much closer to the observed results with *in vitro* translation, whereas the MIC50 is comparable with those of many other antibiotics.

When we first identified MGC-10 as a drug lead, we had hoped it would be effective for systemic use but were disappointed by observed toxicity in mice at a reasonable dose of 10 mg/kg upon IP administration, most likely due to large accumulation in liver, typical of highly lipophilic molecules. The molecule also demonstrated metabolic stability in the microsome stability assays. These results led us to pivot to a topical administration approach to limit systemic exposure. Given the preliminary success with MGC-10 in topical use; however, it may be worth revisiting systemic use at lower doses to explore the potential for finding a therapeutic window.

We observed that mupirocin, although not having a significant effect on dermonecrosis, was able to significantly decrease bacteria counts. Conversely, MGC-10 did not show a statistically significant reduction in bacterial cell count, despite apparent efficacy limiting dermonecrosis, implying that MGC-10 is bacteriostatic in the mouse model. This is in conflict with our *in vitro* data showing that MGC-10 is likely bactericidal in liquid culture. We do not understand the reason for this difference, but it may be related to whether there is any penetration of MGC-10 below the epidermal layers to interact with the injected *S. aureus*, which we did not assess. We also do not know if MGC-10 treatment had any effect on the animals’ immune response to the infection. Regardless, seven different naturally occurring isolates of high-level mupirocin-resistant *S. aureus* were all sensitive to MGC-10, suggesting that MGC-10 would be a good choice for decolonization of mupirocin-resistant skin infections.

Despite the fact that MGC-10 was identified by virtue of its ability to prevent ternary complex formation and was also shown to partially inhibit protein synthesis *in vitro*, it remains possible that its antibacterial activity is due to inhibition of some other cellular target(s). We intend to pursue efforts to provide target validation in the future.

In summary, utilizing a high-throughput screening assay designed to identify inhibitors of ternary complex formation, we have identified a new inhibitor of Gram-positive bacteria, including pathogenic MRSA. This inhibitor, renamed MGC-10, is effective against most if not all known MRSA strains while not harming mammalian cells *in vitro* at concentrations that kill MRSA. Resistance to MGC-10 was not found even after intense efforts to select resistant strains. MGC-10 did show toxicity when used systemically in mice but was not toxic when applied topically, where it was at least as effective as mupirocin (the current topical drug of choice for MRSA) in a mouse skin infection model.

## MATERIALS AND METHODS

### Protocols for high-throughput screening

All solutions were prepared fresh in assay buffer consisting of 70 mM HEPES-KOH (pH7.6), 52 mM ammonium acetate, 8 mM magnesium chloride, 30 mM potassium chloride, and 2.6% glycerol. A 200 nM Cy5-labeled EF-Tu working solution was prepared in assay buffer plus 1.4 mM DTT. An 80 nM Cy3-Phe-tRNA^Phe^ working solution was prepared in an assay buffer containing 20 µM phenylalanine and 2 mM ATP. Solutions were incubated at 37°C for 15–25 min. Three microliters of the Cy5-EF-Tu (or buffer-only control) was dispensed into each well of a black solid 1536-well plate using a Multidrop Combi dispenser (Thermo). Plates were centrifuged for 30 s at 650 × *g* (Eppendorf 5804R). Twenty-three nanoliters of compounds was transferred to the wells using a Kalypsis pin-tool, and the plates were incubated at room temperature and protected from light for 15 min. Then, 3 µL of Cy3-tRNA (or buffer-only control) was dispensed into each well. FRET was then detected using (i) a ViewLux high-throughput microplate imager (PerkinElmer) equipped with Ex: 525/20 and Em: 671/8 filters; or (ii) an Envision Multimode plate reader (PerkinElmer) equipped with Ex: 530/25 and Em: 665 filters.

### Preparation of reagents for qHTS and secondary FRET assays

#### Cy5-EF-Tu

EF-Tu from *E. coli* was expressed and labeled with Cy5 as described previously (3, 5). Sulfonated versions of Cy5 maleimide and Cy3 NHS ester were obtained from GE Healthcare/Amersham or Lumiprobe. Ammonium acetate, magnesium acetate, magnesium chloride, potassium chloride, HEPES, DTT, ATP, GTP, TCEP, and L-phenylalanine were from Sigma.

#### Cy-3-tRNA

tRNA originated from three sources. (i) Work done prior to 2019 (and the quantitative high-throughput screening [qHTS]) was done with purified preparations of *E.coli* tRNA^Phe^ purchased from Chemical Block (Moscow, Russia). This source was no longer available after 2019. (ii) tRNA was prepared from *E. coli* cells that overexpress tRNA^Phe^ according to published procedures (3, 5, 19). (iii) Commercially available preparation of total tRNA (*E. coli* MRE 600) was from Sigma. After labeling and purification, a mix of Cy3-tRNA^Phe^ and Cy3-tRNA^Arg^ was obtained (Fig. S8). The labeling with Cy-3 was previously described (3, 5).

#### Analysis/characterization/quality control (Q)

Before using the reagents in qHTS, Cy3-tRNA and Cy5-EF-Tu preps were checked by UV-Vis spectroscopy, gel electrophoresis, and MALDI-TOF analysis as described previously (3, 5).

### Zone inhibition assay (ZIA)

Strains used to screen for zone inhibition by compounds that scored as hits in the HTS were *B. subtilis* IS-75 and *E.coli* DH5α. The compounds were dissolved in DMSO (Sigma). LB LENOX and LB MILLER media were from IBI (ibisci.com). The 29 highest scoring compounds from LOPAC library were tested, including (R,R)-tetrahydrochrysene from the NCATS group (later, from Biotechne-TOCRIS (Cat. # 1990; www.tocris.com/products/r-r-thc_1990).

An overnight culture of *B. subtilis* was diluted 1/1,000 in LB Miller media and grown up to OD_600_ = 0.1. A 150 µL aliquot of the culture was plated on LB Lenox media Petri dish with 1.5% agar, followed by placing of a 5 mm Whatman filter disk containing 3 µL of 10 mM stock of the tested compound (30 nmoles total) and incubated overnight at 30°C. As controls, we used commercially available disks containing 100 µg carbenicillin or 50 µg ampicillin (from Pfizer).

After the incubation, images of the plates were taken with a smartphone (Fig. S5), and the presence or absence of the inhibition zone was evaluated. In the analysis of MGC-10 analogs (Fig. 7), the diameter of the zone was measured with a ruler. The zone thickness was calculated as 0.5 × (zone diameter less paper disk diameter).

### Inhibition of bacterial growth in liquid culture

Strains and reagents were the same as in the zone-inhibition assay (above). An overnight culture of *B. subtilis* was diluted 1/1,000 in LB Miller media and grown up to OD_600_ = 0.1. The culture was diluted again 1/500 in 1 mL of fresh media with the presence of increasing concentrations of tested compound or 1% DMSO as a control. To calculate CFU, 20 µL aliquots collected in time scale were mixed with 180 µL of saline solution (0.9% NaCl); 100 µL volume of 10 × serial dilutions were prepared, and the samples were plated on LB Lenox media Petri dish with 1.5% agar and incubated overnight.

### Susceptibility of different cell types to inhibition by MGC-10

#### Drug susceptibility assays

The assays were performed at Rutgers Regional Biocontaintment Laboratory (RBL).

Experiments to determine whether MGC-10 inhibits the growth of several bacterial strains, including some pathogens, were performed in 96-well microtiter plates. A DMSO stock solution of each compound was added to the first column and serially diluted across the columns of the plate. The last column of the plate contained no drug and served as a no-drug control. Overnight cultures of the bacterium being tested were diluted 1,000-fold, and 100 µL of the diluted culture (appr. 2 × 10^3^ cells) was used as an inoculum in each well. MIC50s were determined by visual inspection looking for the presence or absence of a pellet after 18 h incubation at 37°C and centrifugation.

#### Cytotoxicity assay

Compounds were tested for cytotoxicity against Vero cells using the CellTiter 96 AQueous One Solution Assay kit (Promega). Vero cells were seeded in 96-well plates at a density of 2 × 10^4^ cells per well, and the plates were incubated for 4 h at 37°C to allow attachments of the Vero cells. Chemical compounds being tested were then added to the wells starting from a final concentration of 50 µg/mL and making 12 1:2 dilutions. Cells were incubated for 72 h at 37°C, after which 20 µL of freshly prepared MTS:PMS reagents were added to each well. Plates were incubated for 2 h and then read at an absorbance of 490 nm.

### Secondary FRET assay

An EnVision 2104 Multilabel Plate Reader (PerkinElmer) was used with an excitation optical filter 2100–5050 Bodipy TMR FR 531 nm and emission optical filter 2100–5770 Cy5 685 nm. Assay was performed as previously described (3, 5).

### Counterscreen assay aimed at excluding quenching as a mechanism of inhibition of FRET

Oligonucleotides were purchased from Integrated DNA Technologies: (i) CTC TGG GAA CAT CCT /3`Cy5Sp/ and (ii) /5`Cy3/TT TTT AGG ATG TTC CCA GAG.

In the assay, we used 50 nM final concentration of each oligonucleotide.

The incubation buffer was 70 mM HEPES-KOH (pH 7.6), 52 mM ammonium acetate, 8 mM magnesium chloride, 30 mM potassium chloride, and 2.6% glycerol. We used a 96-well black, clear bottom microplate, and read the plate using EnVision multilabel plate reader (PerkinElmer) having the same optical filters and parameters as described before. The assay was performed as described above (Fig. S6).

### Purified *in vitro* translation assay

PURExpress *in vitro* purified ribosomal translation kit (New England Biolabs) was used. Biochemical protocols used were as described by the manufacturer.

### MGC-10 ointment formulation and preparation

Ointment preparation was based on information from the University of North Carolina-Eshelman School of Pharmacy website (https://pharmlabs.unc.edu/labexercises/compounding/ointments/). 250 µg of stearyl alcohol (Sigma-Aldrich) and 250 µL of white petrolatum (Walgreens) were melted and mixed on a 70°C hot plate. Separately, 100 µL of 10% sodium dodecyl sulfate (SDS; Sigma-Aldrich) was mixed with 120 µL of propylene glycol (Sigma-Aldrich) and 280 µL Milli-Q water. The water phase was added by drops to the oleaginous phase, pipetting each drop from a 1 mL glass syringe preheated to 70°C. Using a 1 mL disposable syringe, 500 µL of suspension was removed and saved as a negative control. To the remaining volume, 50 µL of 200 mg/mL MGC-10 in ethanol was added. The preparation was mixed well to form a homogeneous suspension. One milliliter was removed with a disposable syringe, which was then used as a preparation of MGC-10 in ointment.

### Mouse skin infection model

Animal work at Rutgers in this study was conducted in strict accordance with the recommendations in the Guide for the Care and Use of Laboratory Animals of the NIH, the Animal Welfare Act, and US federal law. Protocols were approved by the Institutional Animal Care and Use Committee of Rutgers New Jersey Medical School of Newark, New Jersey, USA.

Six-week-old female C57Bl/6 J mice were purchased from Jackson Laboratories. Mice were infected subcutaneously with 2 × 10^7^ CFU of *S. aureus* USA300 (20), prepared in 100 µL phosphate buffered saline (PBS) to the shaved backs of mice while under anesthesia (ketamine/xylazine). Five minutes after inoculation, 50 µL of ointment (1 mg of drug) was applied to the regions of inoculation. Ointment was heated to 37°C for 20 min to improve the consistency for application to mouse skin. The ointment was applied daily. Regions of dermonecrosis were measured daily. The experiment was stopped after day 5. Mice were euthanized through carbon dioxide and confirmed through cervical dislocation. In total, 5 mm punch biopsies were collected from the dermonecrotic lesion and homogenized to determine bacterial burden through serial dilution on chromogenic media (*S. aureus* chromagar, BD Biosciences). Supernatant samples were saved for future use. Topical mupirocin, 20 mg/mL, was from Glenmark Pharmaceuticals.

### Time-kill experiments with *S. aureus*

*S. aureus* USA300 was inoculated 1:100 into LB medium from overnight cultures. Cells were grown to the early exponential phase and back diluted to OD_600_ 0.1. Cultures were treated with 15 µM MGC-10 or DMSO and sampled at 0, 1, 2, and 4 h. Samples were serially diluted and plated on LB agar. Colonies were enumerated after overnight incubation at 37°C. Time-kill experiments were performed with *n* = 4 biological replicates.

### MIC testing of *S. aureus* and other staphylococcus strains

Bacterial strains were sub-cultured from frozen glycerol stocks and grown on blood agar overnight at 37°C. The following day, they were resuspended in physiological saline and normalized to OD_600_ = 0.2 using a spectrophotometer. Normalized mixtures were diluted 1:500 and subjected to MIC susceptibility testing using broth microdilution against MGC-10 concentrations ranging from 0 to 50 µM. Microtiter plates were incubated for 16 h at 37 C, and then assessed the following day to determine the minimum inhibitory concentration.

### Attempts to generate mutants resistant to MGC-10 in *S. aureus*

We attempted to identify a mutant resistant to MGC-10 using two different approaches. MRSA clinical isolates typed as USA300 and USA500 with an MIC of 6 µM to MGC-10 were grown overnight in sub-MIC concentrations in LB and then plated on LB-agar gradient plate that ranged in MGC-10 concentration from 0 to 12 µM. All identified colonies were retested at the same MIC as the parent strain. The second approach screened a Himar1 transposon library (21), which we constructed in laboratory *S. aureus* strain 8325 with an MGC-10 MIC of 12 µM. The Tn-library was plated at 25 µM MGC-10 with the goal of identifying a loss-of-function mutation that yields resistance.

### Protocols for stability of MCG-10 *in vitro* with liver microsomes

#### Multi-species multi-time point microsome stability assay

Briefly, each reaction mixture (110 µL) consisted of a test article (1 µM), mix gender human, male Sprague Dawley rat or male CD-1 mouse microsomal fractions (0.5 mg/mL), and NADPH regenerating system in phosphate buffer at pH 7.4. Samples were incubated in 384-well plates at 37°C for 0, 5, 10, 15, 30, and 60  min. Sample analysis and half-life calculations were performed using a previously described method (22). The three controls used were Buspirone (short half-life), Loperamide (moderate half-life), and Antipyrine (long half-life).

#### Microsomal stability categories

Categories were defined as follows: unstable, t_1/2_ < 30 min; stable, t_1/2_ > 30 min.

### Pharmacokinetics protocol

Male C57B6 mice, 3 mice per time point, were subjected to intraperitoneal injection (IP) at 10 mg/kg MGC-10 in a solution of 30% Solutol, 30% polyethylene glycol 300, and 40% (40% HP-b-CD). Plasma, lung, brain, liver, and skin samples were collected over 168 h. The concentrations of MGC-10 were determined by ultra-performance liquid chromatography-tandem mass spectrometry (UPLC-MS/MS) bioanalytical method, except for skin.

### UPLC-MS/MS method to quantify MGC-10

Samples were prepared as follows: protein was precipitated in 96-well plates for plasma and tissue homogenate samples. In total, 10 µL plasma or tissue homogenate samples were mixed with 200 µL of acetonitrile (ACN) with internal standard. Plates were vortexed and centrifuged at 4°C. 150 µL supernatant was transferred to injection plates. Additionally, 1.0 µL was injected for UPLC-MS/MS analysis. A Waters Xevo TQ-S triple quadruple mass spectrometer was used, as well as a Waters Acquity UPLC BEH C18, 1.7 µm, 2.1 × 100 mm column. The mobile phase flow rate was 0.6 mL/min (mobile phase A: 0.1% formic acid in H2O and mobile phase B: 0.1% formic acid in ACN) with the gradient method. Standards (STDs) and quality controls (QCs) were prepared in a control blank matrix of plasma, brain, liver, and lung.

## SUPPLEMENTAL MATERIAL

Figure S1 aem.02046-24-s0001.tiff Comparison of Envision versus ViewLux multiwell plate readers.

Figure S2 aem.02046-24-s0002.tiff Assay components are stable at room temperature for at least 4 hours.

Figure S3 aem.02046-24-s0003.tiff Compound inhibition profiles.

Figure S4 aem.02046-24-s0004.tiff Kirromycin effect on FRET in the manual (offline) versus automated (online) assays.

Figure S5 aem.02046-24-s0005.tiff Images of representative plates at the end of the ZIA.

Figure S6 aem.02046-24-s0006.tiff FRET (vertical axis) for different combination of reagents.

Figure S7 aem.02046-24-s0007.tiff Concentration-time profiles after IP administration of 10 mg/kg MGC-10 in mice.

Figure S8 aem.02046-24-s0008.tiff Separation of Cy3-labeled tRNA using reverse phase HPLC.

Supplemental legends aem.02046-24-s0009.docx Legends for Fig. S1 to S8.

ASM does not own the copyrights to Supplemental Material that may be linked to, or accessed through, an article. The authors have granted ASM a non-exclusive, world-wide license to publish the Supplemental Material files. Please contact the corresponding author directly for reuse.
