# Supplementary figures and images for: Identification of an antibiotic from an HTS targeting EF-Tu:tRNA interaction: a prospective topical treatment for MRSA skin infections
Source: Appl Environ Microbiol. 2024 Dec 23;91(1):e02046-24. doi: 10.1128/aem.02046-24 (PMC11784183; doi:10.1128/aem.02046-24)

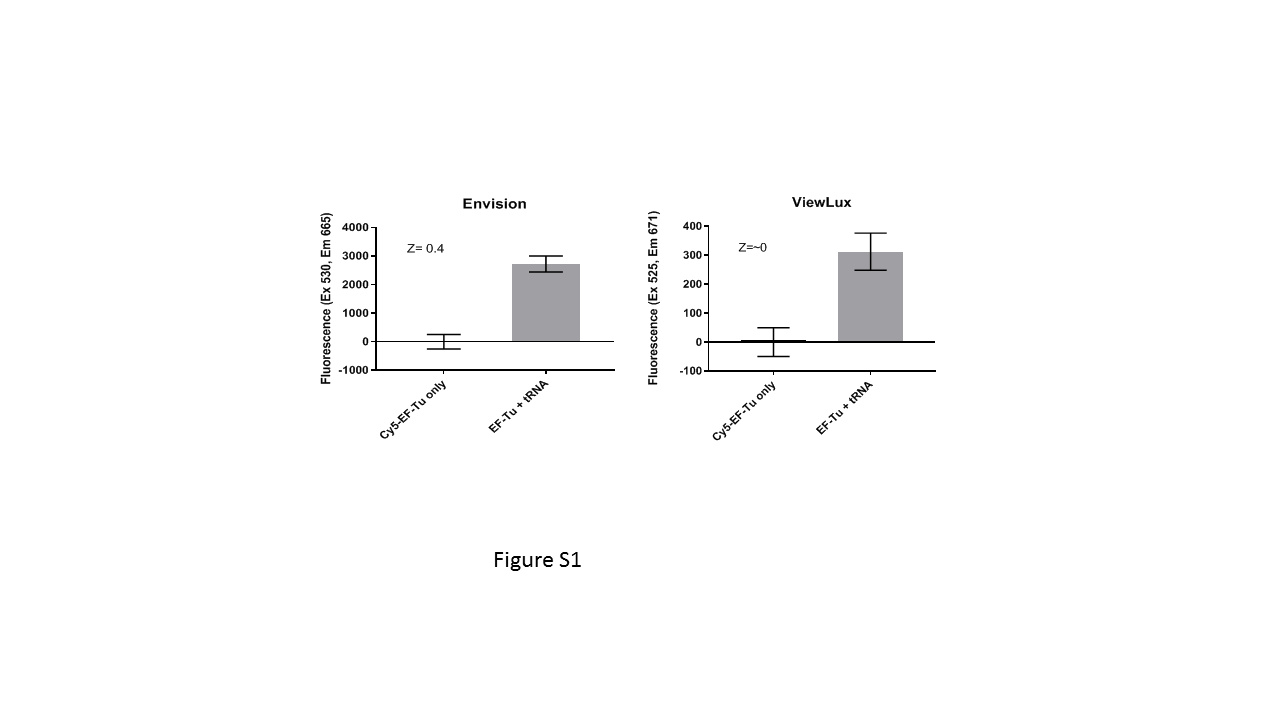

Supplement: Figure S1 — Comparison of Envision versus ViewLux multiwell plate readers. [file aem.02046-24-s0001.tiff]

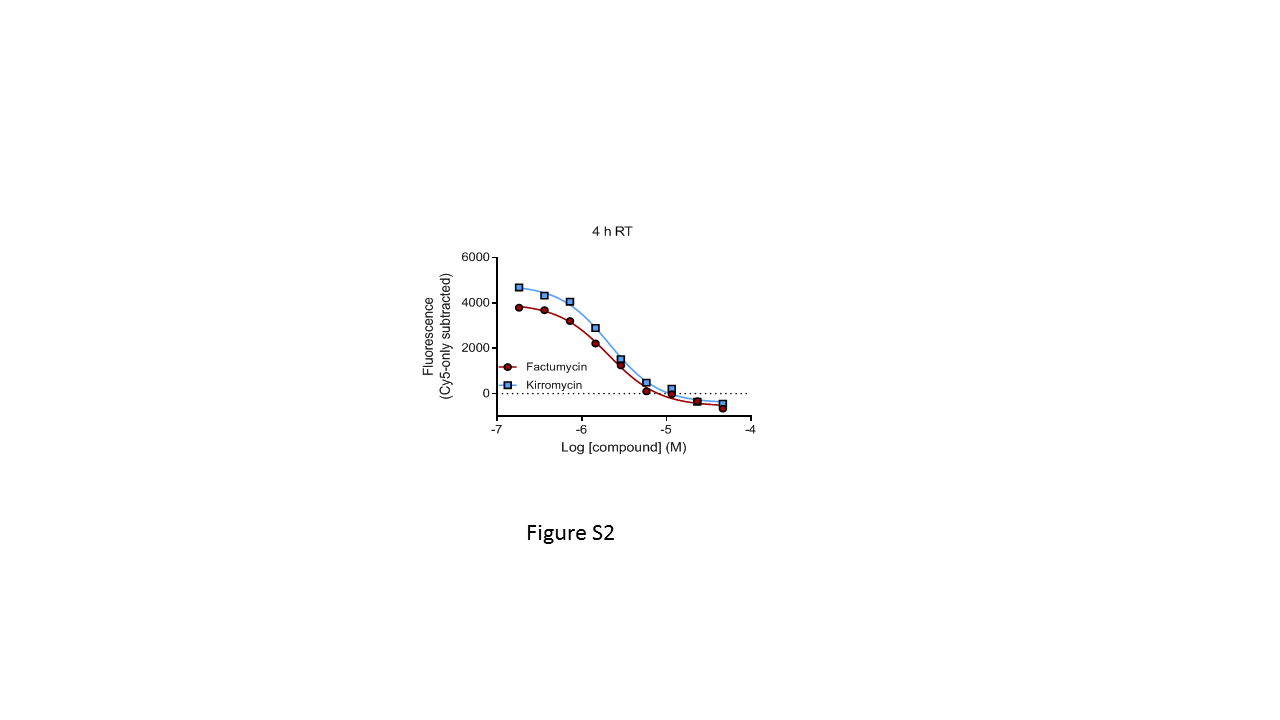

Supplement: Figure S2 — Assay components are stable at room temperature for at least 4 hours. [file aem.02046-24-s0002.tiff]

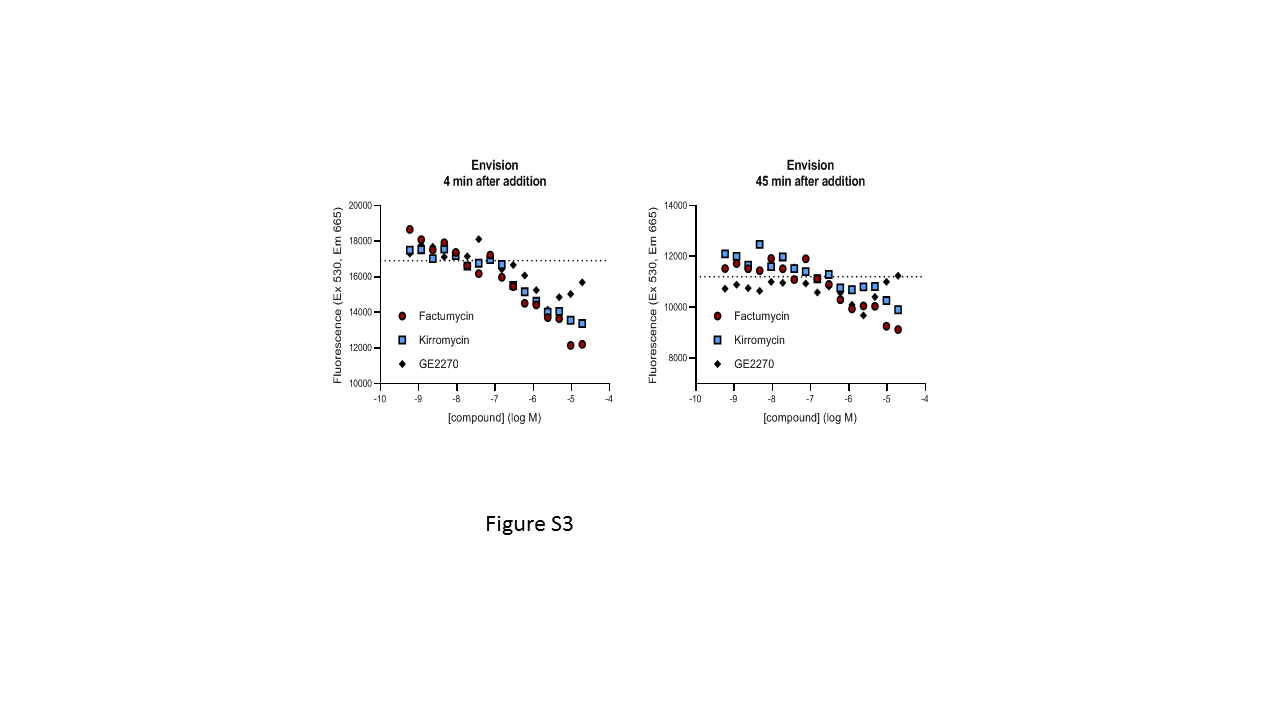

Supplement: Figure S3 — Compound inhibition profiles. [file aem.02046-24-s0003.tiff]

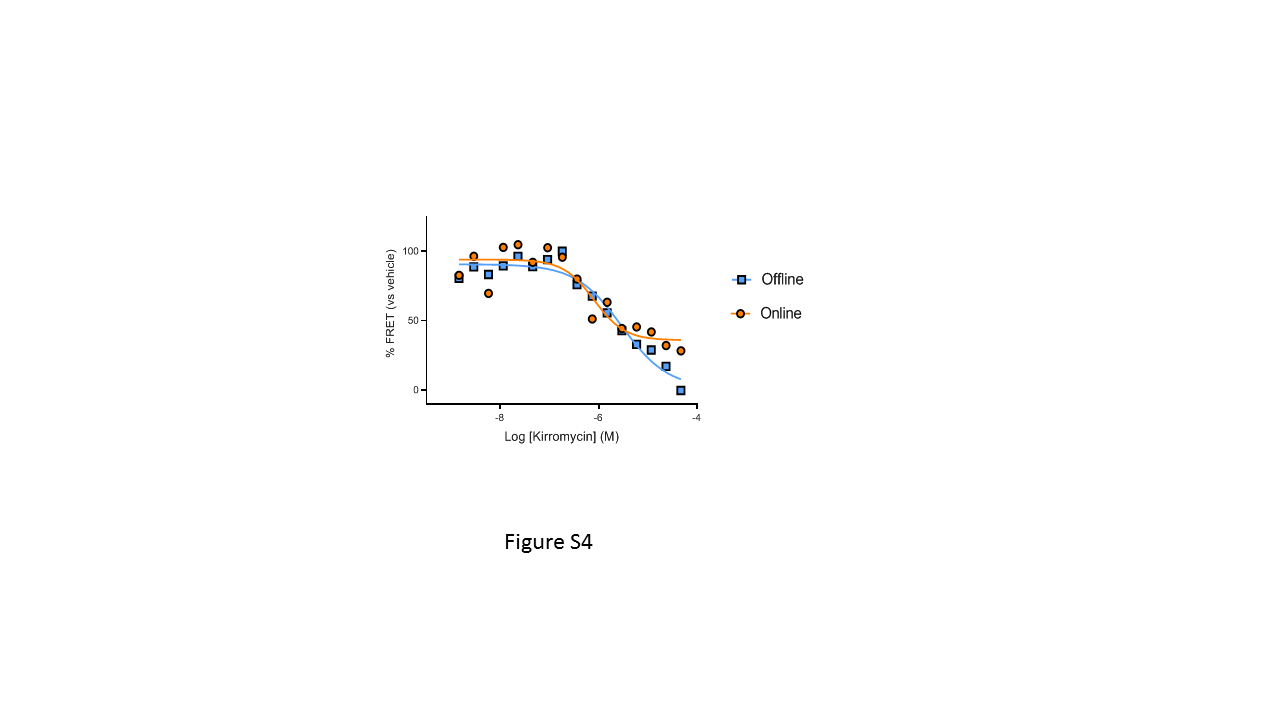

Supplement: Figure S4 — Kirromycin effect on FRET in the manual (offline) versus automated (online) assays. [file aem.02046-24-s0004.tiff]

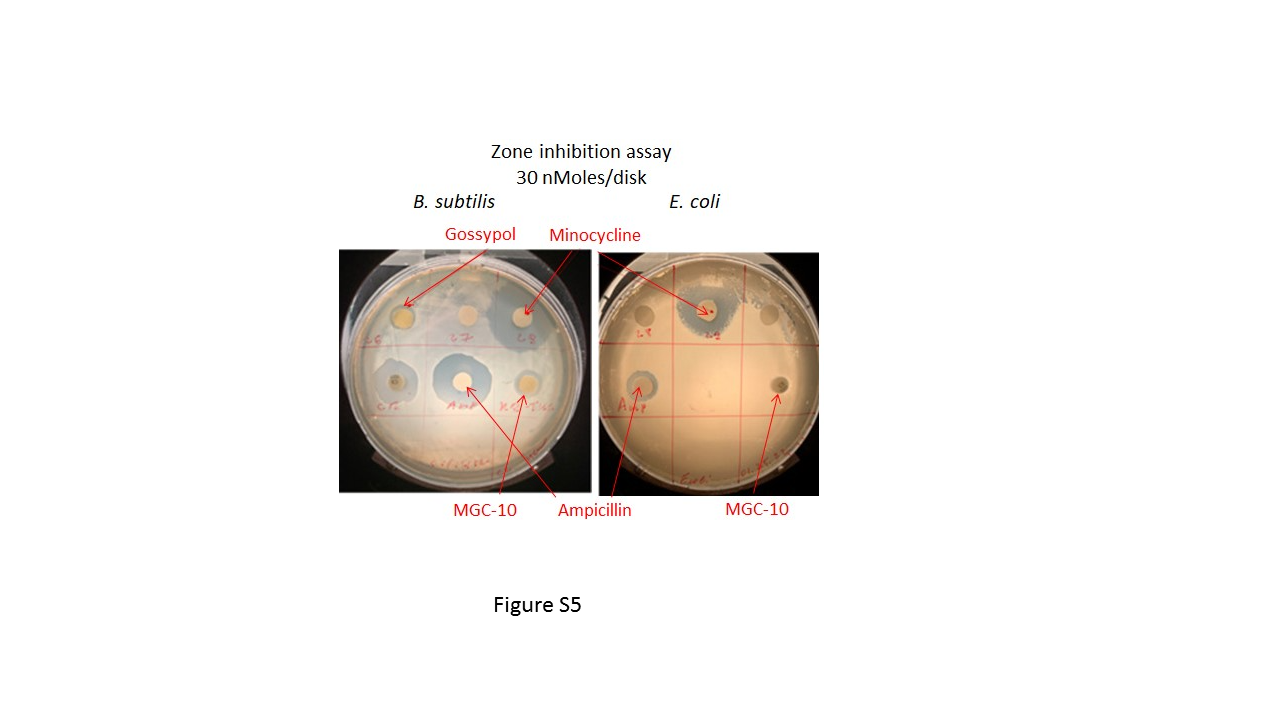

Supplement: Figure S5 — Images of representative plates at the end of the ZIA. [file aem.02046-24-s0005.tiff]

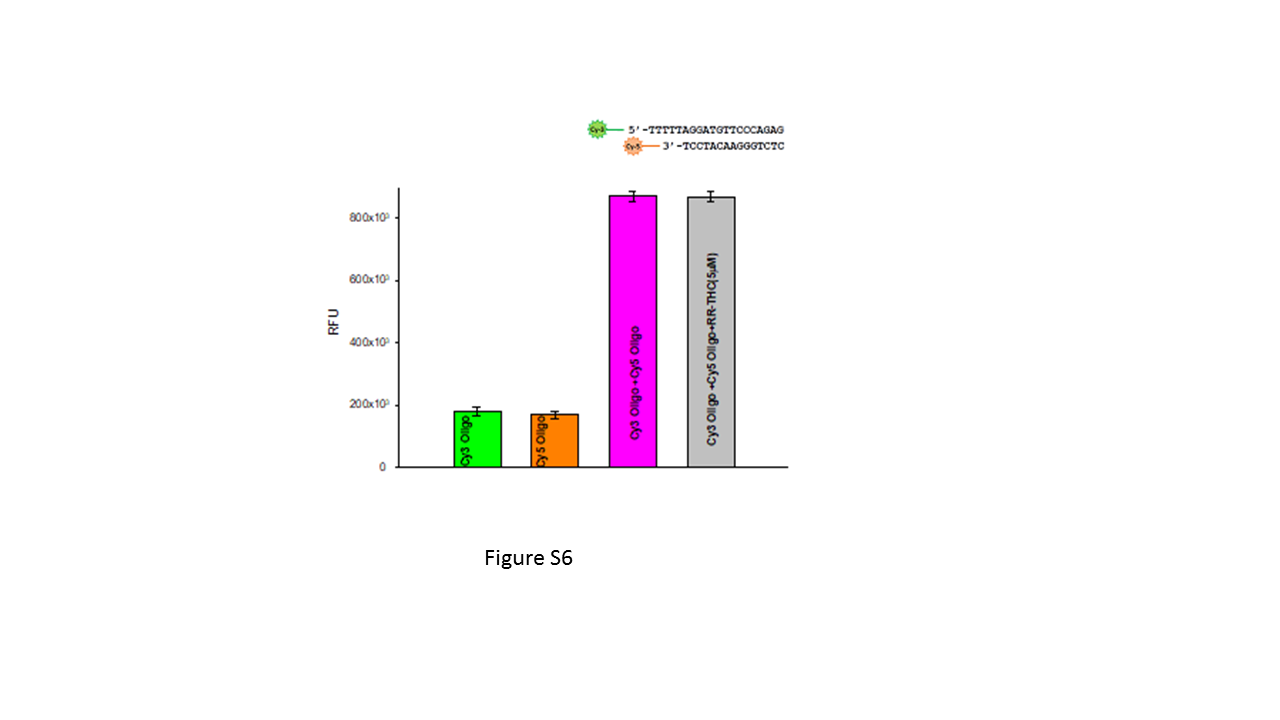

Supplement: Figure S6 — FRET (vertical axis) for different combination of reagents. [file aem.02046-24-s0006.tiff]

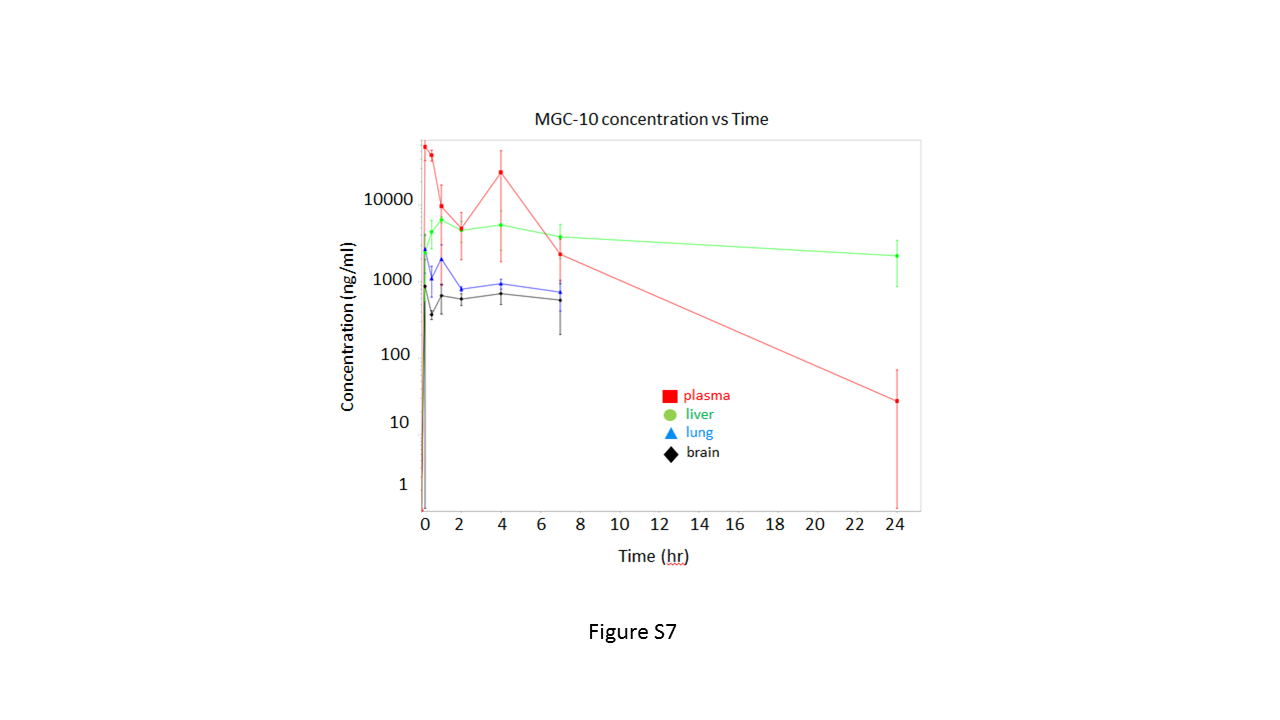

Supplement: Figure S7 — Concentration-time profiles after IP administration of 10 mg/kg MGC-10 in mice. [file aem.02046-24-s0007.tiff]

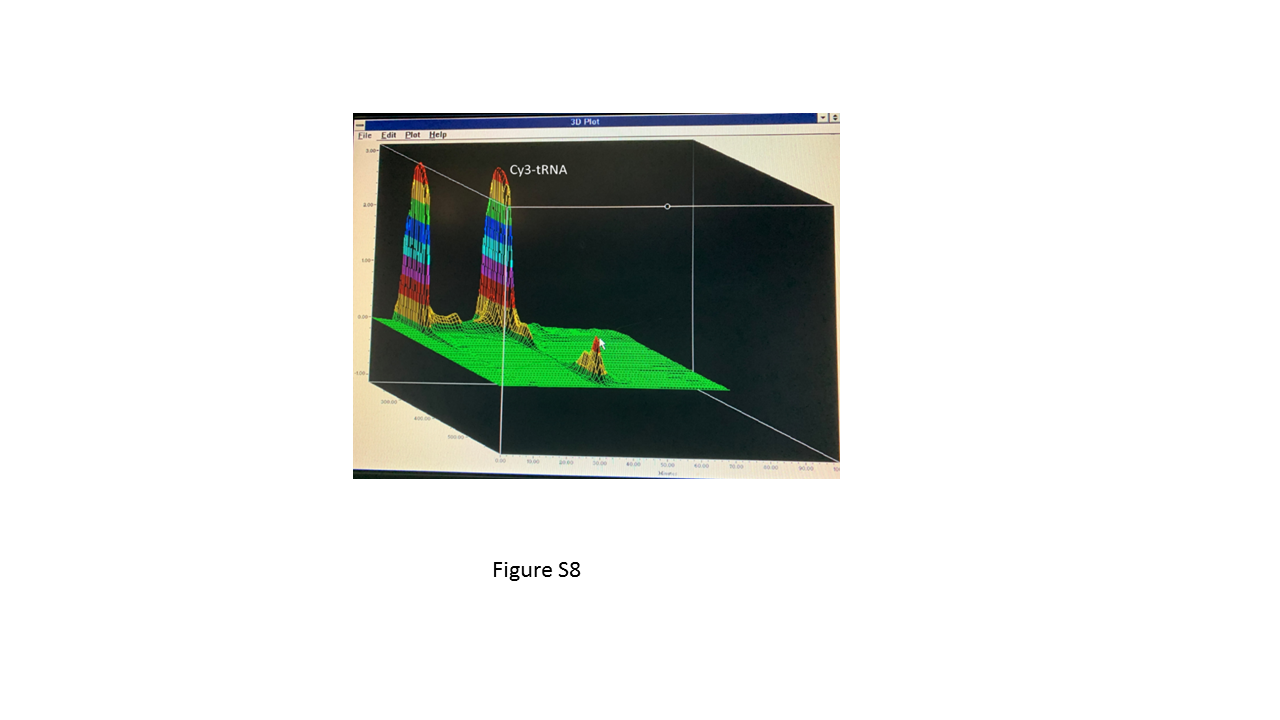

Supplement: Figure S8 — Separation of Cy3-labeled tRNA using reverse phase HPLC. [file aem.02046-24-s0008.tiff]
