# Supplementary material for: Identification of an antibiotic from an HTS targeting EF-Tu:tRNA interaction: a prospective topical treatment for MRSA skin infections
Source: Appl Environ Microbiol. 2024 Dec 23;91(1):e02046-24. doi: 10.1128/aem.02046-24 (PMC11784183; doi:10.1128/aem.02046-24)
Supplement: Supplemental legends — Legends for Fig. S1 to S8. [file aem.02046-24-s0009.docx]

**LEGENDS TO FIGURES IN SUPPLEMENTARY MATERIALS**

Figure S1: Comparing Envision versus ViewLux multiwell plate readers.

Figure S2: Assay components are stable at room temperature to at least 4 hours. Shown is a representative experiment which was repeated.

Figure S3: Compound Inhibition Profiles. FRET was captured either 4 min or 45 min after addition of all assay components. The DMSO control value (average from 32 wells) is indicated by the dotted line. Shown are representative experiments which were repeated.

Figure S4: Kirromycin effect on FRET in the manual (offline) versus automated (online) assays.

Figure S5. Images of representative plates at the end of the ZIA.

Figure S6. FRET (vertical axis) for different combination of reagents. Left two bars: two oligos separately. Third and fourth bars from left: fluorescence of the duplex DNA in the absence and presence of MGC-10, respectively. The presence of MGC-10 did not significantly change FRET suggesting that the effect of MGC-10 on fluorescence in qHTS is minimal if any (i.e., no quenching).

Figure S7: Concentration-time profiles after IP administration of 10 mg/kg MGC-10 in mice.

Figure S8**.** Separation of Cy3-labeled tRNA using reverse phase HPLC. The chromatogram on the left side: unlabeled tRNA. Chromatogram labeled Cy3-tRNA: note small peak(s) in the center corresponding attached Cy3 Dye. Preparative C4 250x21 mm column from Kromasil was used for separation.
